# Interfacial Adhesion Property of Asphalt Binder with Calcium Alginate Carrier of Asphalt Rejuvenator

**DOI:** 10.3390/molecules28114447

**Published:** 2023-05-30

**Authors:** Yan Li, Bowei Sun, Zirui Wu, Lina Wang, Xiaogang Guo

**Affiliations:** 1 School of Civil Engineering and Architecture, Nanyang Normal University, Nanyang 473061, China; 2 School of Transportation Science and Engineering, Civil Aviation University of China, Tianjin 300300, China; 3 School of International Education, Henan University of Science and Technology, Luoyang 471000, China; 4 School of Water Conservancy Engineering, Zhengzhou University, Zhengzhou 450001, China; 5 School of Concrete and Construction Management, Middle Tennessee State University, Murfreesboro, TN 37132, USA

**Keywords:** interfacial adhesion, asphalt binder, calcium alginate carrier, molecular dynamics simulation, relative concentration, interfacial adhesion work

## Abstract

Recently calcium alginate has been successfully applied to encapsulate asphalt rejuvenator, which can protect asphalt rejuvenator from early leakage and release asphalt rejuvenator when triggered by specific factors such as cracks. The interfacial adhesion property of asphalt binder with calcium alginate carrier is of great importance to its actual performance. In this paper, the molecular model of the interface region between asphalt binder and calcium alginate was established, and molecular dynamics simulations were performed on it to investigate the molecular interaction at the interface region. By extracting and processing the data during the simulation process, the interfacial adhesion behavior was expounded using the spreading coefficient (S), permeation depth and permeation degree. Furthermore, the interfacial adhesion strength was evaluated by adopting the interfacial adhesion work. Results showed that the value of S was greater than 0, implying that asphalt binder could wet the surface of calcium alginate. Saturate had the highest value of permeation degree, followed by resin, aromatic and asphaltene. However, asphalt binder could not infiltrate into the interior of TiO_2_, only accumulating and spreading on the surface of TiO_2_. The interfacial adhesion work of unaged and aged asphalt binder to calcium alginate was −114.18 mJ/m^2^ and −186.37 mJ/m^2^, respectively, similar to that of asphalt–aggregate interface. The van der Waals interactions contributed the most to the formation of the interfacial adhesion strength. In addition, a certain degree aging of asphalt binder and addition of titanium dioxide in the calcium alginate carrier were helpful to enhance the interfacial adhesion strength.

## 1. Introduction

Sodium alginate consisting of α-l-guluronic acid (G-block) and β-d-mannosyl acid (M-block) is a natural macromolecule with high viscosity [1]. The free carboxyl group in sodium alginate is easy to cross-link with divalent metal cations to form water-insoluble alginate gels. Usually, calcium ion is used to produce calcium alginate that has been widely applied in many fields such as food, textile and biomedicine due to its advantages of high water absorption ability, excellent film-forming ability, three-dimensional porous network structure, environmental friendliness, etc. In recent years, calcium alginate with porous structure has been gradually introduced to road engineering as a promising approach for enhancing the self-healing performance of asphalt pavement [2,3,4,5,6,7]. Specifically, it is used to encapsulate asphalt rejuvenator, such as commercial rejuvenators and vegetable oils, and then embedded in asphalt pavement to accelerate the self-healing behavior of asphalt binder. That is to say, calcium alginate serves as a carrier for asphalt rejuvenator. During the service period of asphalt pavement, asphalt rejuvenator will be released from calcium alginate carrier as designed when stirred by vehicle loadings or pierced by cracks occurred in the vicinity of the carrier. The released asphalt rejuvenator flows into the cracks, permeates into the surrounding asphalt binder, and ultimately recovers the property of aged asphalt binder and accelerates the crack healing process [8].

Calcium alginate carrier has attracted close attention from road engineers due to its excellent performance. It was produced with various shapes and sizes, including the millimeter-sized capsule, micrometer-sized capsule, and micrometer-sized fiber. Tabakovi et al. firstly explored the potential application of calcium alginate as a carrier for asphalt rejuvenator using a wet spinning plant in 2016 and demonstrated that the calcium alginate fiber containing asphalt rejuvenator had enough mechanical strength and high-temperature stability to successfully survive the construction process of asphalt pavement [9]. Micaelo et al. investigated the influence of millimeter-sized capsules containing sunflower oil on the mechanical property and self-healing ability of asphalt mixture [2]. They proposed that the capsules showed positive effects on the crack healing ability of asphalt mixture but decreased the deformation resistance of the asphalt mixture. Norambuena-Contreras et al. conducted tests to investigate the effect of the content of calcium alginate capsule containing rejuvenator on the performance of dense-graded asphalt mixtures and proposed that the optimal content was 0.5% by weight of asphalt mixture [10]. Zhang et al. also reported that the content of 0.5% was the most appropriate in view of the comprehensive performance of asphalt mixture [11]. Al-Mansoori et al. demonstrated that calcium alginate capsules containing rejuvenator could meet the strict construction requirements of asphalt pavements and significantly improve the self-healing properties of asphalt mixtures [12,13,14]. Norambuena-Contreras et al. suggested that calcium alginate capsules produced by an injection pump showed better shape regularity and mechanical strength than the funnel fabrication method [15]. Shu et al. synthesized calcium alginate microcapsules and calcium alginate fibers using microfluidic devices and studied the regulation method of microcapsule size [16,17].

According to the published research [9,10,11,12,13,14,15,16,17], the calcium alginate carrier can successfully encapsulate asphalt rejuvenator, survive the construction period of asphalt pavement, and release asphalt rejuvenator when triggered by cracks during the service period. Most of the research focuses on the fabrication techniques, characterization methods and influences on the performance of asphalt materials. The interfacial adhesion behavior of calcium alginate carrier with asphalt binder plays a vital role in the working process [18]. Strong interfacial adhesion allows the calcium alginate carrier to bond tightly with the asphalt mixture, thus ensuring effective load transfer from asphalt matrix to the carrier to trigger the release of rejuvenator as designed. However, there are few published reports focused on the subject of interfacial adhesion property whose characteristics, mechanism and evaluation methods are still unclear [4,18].

The molecular dynamics (MD) simulation method has been proven to be an effective tool for investigating the interfacial adhesion behavior of asphalt binder with other materials at the nanoscale [19,20]. In this study, the MD simulation method was adopted to investigate the interfacial adhesion behavior between calcium alginate carrier and asphalt binder at the molecular scale, aiming to explore its intrinsic characteristics and evaluating the interfacial adhesion performance. First, the molecular model of the interfacial adhesion was established using Materials Studio 7.0 (MS 7.0) software. Then, the molecular dynamics simulations were conducted on the molecular model to make it reach a thermodynamic equilibrium state. Next, the data recorded during the simulation process was extracted and processed to analyze the molecular interaction characteristics at the interface region. Finally, the interfacial adhesion strength was evaluated using the interfacial adhesion work.

## 2. Result and Discussion

### 2.1. Analysis of Interfacial Adhesion Behavior

The interfacial adhesion behavior between asphalt binder and calcium alginate carrier can be mainly interpreted from two aspects: (1) the interface wetting process of asphalt binder along the surface of calcium alginate; and (2) the permeation process of asphalt binder into the interior of calcium alginate. In this section, the molecular interaction between shell material and asphalt binder at the interface region is analyzed from the abovementioned two aspects.

#### 2.1.1. The Interfacial Wetting Process

The interfacial wetting ability depends on the relative magnitude of the cohesion work (intermolecular attraction among asphalt molecules) and the adhesion work (intermolecular attraction between asphalt molecules and shell molecules). Only when the adhesion work is greater than the cohesion work, asphalt binder can spread on the surface of the shell material, that is, it can wet the shell surface. The differential value of the adhesion work and the cohesion work is defined as the spreading coefficient *S* whose calculation method is shown in Equation (1).

(1) $$S=\left|W_{adhesion}\right|-\left|W_{cohesive}\right|$$

where: *S* is the spreading coefficient; *W_adhesion_* is the adhesion work for the interface of asphalt binder and shell material; *W_cohesive_* is the cohesion work of asphalt binder.

The values of *W_adhesion_* and *W_cohesive_* were obtained by extracting simulation data from the equilibrium configuration of the interfacial adhesion models, and thus the value of *S* could be calculated, as listed in Table 1. It can be seen that the absolute value of *W_adhesion_* is greater than that of *W_cohesive_*, demonstrating that asphalt binder can wet the surface of shell material. Moreover, the value of *S* for asphalt binder on the TiO_2_ surface is greater than that for asphalt binder on the surface of calcium alginate. This means that it is easier for asphalt binder to spread on the TiO_2_ surface. The effect of asphalt aging on the interfacial wetting process is bidirectional. On one hand, the absolute value of *W_cohesive_* increases from 80.26 mJ/m^2^ to 83.41 mJ/m^2^ after the oxidative aging of asphalt binder, which indicates that the oxidative aging enhances the molecular interaction among asphalt molecules. On the other hand, the value of *W_adhesion_* of asphalt binder to calcium alginate and titanium dioxide increases from 114.18 mJ/m^2^ and 205.82 mJ/m^2^ to 186.37 mJ/m^2^ and 210.87 mJ/m^2^, respectively. After the oxidative aging of asphalt binder, the increasing amount of *W_adhesion_* is greater than that of *W_cohesive_*, especially for the interfacial adhesion model of asphalt binder and calcium alginate. Therefore, the aging degree of asphalt binder simulated in this study can promote the interfacial wetting process between asphalt binder and shell material.

#### 2.1.2. The Permeation Process

**Figure 1:**
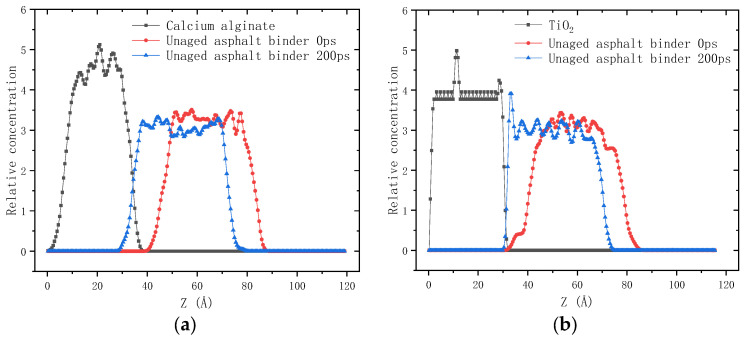
Relative concentration distribution of molecules along the z-axis in the interfacial adhesion model: (**a**) Calcium alginate and unaged asphalt binder; (**b**) TiO_2_ and unaged asphalt binder.

Figure 1 shows the relative concentration distribution of asphalt molecules along the *z*-axis before and after the 200 ps dynamics simulation. Relative concentration is defined as the ratio of the atomic density per unit length along the axis to the average atomic density of the amorphous cell. Calcium alginate and titanium dioxide are solid at room temperature, whose spatial positions are fixed during the simulation process.

As shown in Figure 1a, in the initial configuration of the interfacial adhesion model of calcium alginate and asphalt binder, there are three main regions: the left region filled with calcium alginate molecules (0–40 Å), the middle region filled with asphalt molecules (40–85 Å), and the right vacuum region (85–120 Å). After the 200 ps dynamics simulation, asphalt molecules moved left along the *z*-axis, reaching the maximum relative concentration on the interface (40 Å). It could be inferred that there is a strong intermolecular interaction between calcium alginate molecules and asphalt molecules, allowing asphalt molecules to adsorb on the surface of calcium alginate. Due to the abundant microvoids in calcium alginate, asphalt molecules further penetrated into the interior of calcium alginate, forming a good interfacial interaction effect.

As shown in Figure 1b, in the initial configuration of the interfacial adhesion model of TiO_2_ and asphalt binder, the TiO_2_ molecules are distributed in the region of 0–30 Å, while asphalt molecules are in the region of 30–85 Å, and the vacuum layer is in the region of 85–115 Å. After the 200 ps dynamics simulation, asphalt molecules moved toward the titanium dioxide layer, reaching the maximum relative concentration at the interface. The surface of TiO_2_ is smooth and dense, making it very hard for asphalt molecules to infiltrate into its interior. Consequently, asphalt molecules accumulated and spread on its surface, resulting in a significant increase in the relative concentration of asphalt molecules on its surface compared with that on the surface of calcium alginate.

**Figure 2:**
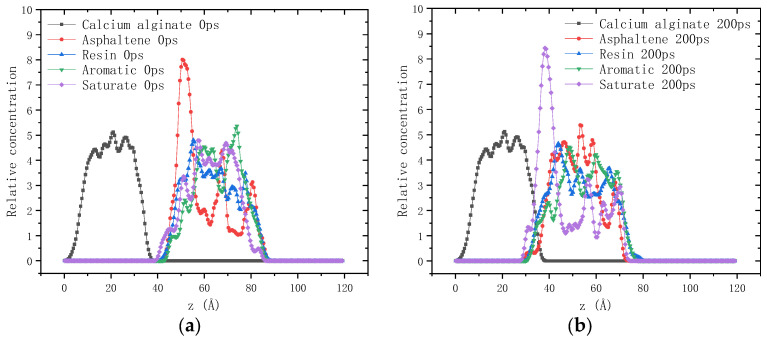
Relative concentration distribution of molecules along the z-axis in the interfacial adhesion model of calcium alginate and asphalt binder: (**a**) 0 ps; (**b**) 200 ps.

**Figure 3:**
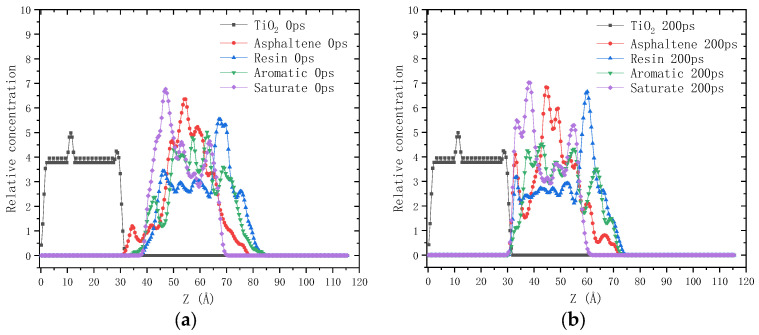
Relative concentration distribution of molecules along the z-axis in the interfacial adhesion model of TiO_2_ and asphalt binder: (**a**) 0 ps; (**b**) 200 ps.

The relative concentration distribution of each component along the z-axis before and after the 200 ps dynamics simulation is drawn in Figure 2 and Figure 3. It can be seen that after the 200 ps dynamics simulation, the molecules of the four components of asphalt binder all moved towards the shell material (calcium alginate and TiO_2_), which is consistent with the overall movement of asphalt molecules. Nevertheless, it should be noted that the moving distance varies significantly among the four components. In order to quantitatively characterize the permeation behavior of asphalt molecules into calcium alginate, two indexes were proposed in this study: permeation depth and permeation degree. The former index refers to the maximum diffusion distance (Å) of asphalt molecules permeating into calcium alginate at equilibrium state. The latter is defined as the integral of the relative concentration distribution curve of asphalt molecules at equilibrium state (200 ps) in the range of permeation depth. Their calculation results are shown in Table 2.

In the interfacial adhesion model of calcium alginate and unaged asphalt binder, the permeation depth and permeation degree of saturate is the largest compared with that of the other three components. This can be attributed to its small molecular weight and slender molecular structure, endowing it with strong diffusion ability. The permeation depth and permeation degree of resin are slightly higher than those of aromatic, which can be explained by the higher polarity of resin strengthening its interaction with calcium alginate. Asphaltene has the largest molecular weight and the slowest diffusion ability, so its permeation depth and permeation degree are the lowest. For the aged asphaltene, resin and aromatic, the oxygen-containing functional groups increase, leading to their higher molecular polarity and stronger interaction with calcium alginate. Consequently, their permeation depth and permeation degree all increase greatly. The permeation depth and permeation degree of saturate is only slightly increased, which might be correlated to the push by other components during the dynamics simulation period. Asphalt molecules cannot penetrate into the titanium dioxide, so the values of permeation depth and permeation degree are both zero. It can be concluded that the compactness of calcium alginate carrier will be improved by adding a certain amount of titanium dioxide into the shell, which helps to prevent the premature contact and interaction between asphalt binder with asphalt rejuvenator encapsulated in calcium alginate carrier.

### 2.2. Evaluation of Interfacial Adhesion Strength

The interfacial adhesion energy of shell material and asphalt binder refers to the work required to peel asphalt binder from the surface of calcium alginate carrier, whose calculation method is shown in Equation (2). To avoid the influence of the interface contact area, the interfacial adhesion work (*W_adhesion_*), defined as the work required for interface peeling per unit area, is used in this study to evaluate the resistance of asphalt binder to be peeled from the shell material [21,22]. It can be obtained using Equation (3). When the value of *W_adhesion_* is positive, it indicates that the materials on the two sides of the interface repel each other. In contrast, the negative value of *W_adhesion_* indicates that the materials are attracted to each other. The greater the absolute value of *W_adhesion_* is, the stronger the interaction force between the two materials, and the higher the interfacial adhesion strength is.

(2) $$\Delta E_{adhesion}=E_{total}-\left(E_{f}+E_{a}\right)$$

(3) $$W_{adhesion}=\frac{\Delta E_{adhesion}}{A}\times K$$

where: $\Delta E_{adhesion}$ represents the interfacial adhesion energy of shell material and asphalt binder (kcal/mol); *E_total_* represents the total potential energy of the interfacial adhesion model at equilibrium state (kcal/mol); $E_{f}$ represents the potential energy of the shell material at equilibrium state (kcal/mol); $E_{a}$ represents the potential energy of asphalt binder at equilibrium state (kcal/mol); *W_adhesion_* represents the interfacial adhesion work of shell material and asphalt binder (mJ/mol); A represents the interface contact area (Å^2^); and *K* represents the unit conversion coefficient, *K* = 695.

The calculation results of the interfacial adhesion energy/work are listed in Table 3. The calculated results are all negative, indicating the mutual attraction between shell material (calcium alginate/titanium dioxide) and asphalt binder. The contribution value of bonding interaction energy (∆*E_valence_*) to ∆$E_{adhesion}$ is zero for the four interfacial adhesion models, demonstrating that there is no chemical reaction. The absolute value of van der Waals interaction energy (∆*E_vdW_*) is much larger than that of electrostatic interaction energy (∆*E_elec_*), which means that Van der Waals interactions contribute the most to the formation of interfacial adhesion. It is reported that the absolute value of interfacial adhesion work of asphalt binder to aggregate mainly consisting of silicon dioxide, is in the range of 100–180 mJ/m^2^ [23]. The interfacial adhesion work of unaged and aged asphalt binder to calcium alginate is −114.18 mJ/m^2^ and −186.37 mJ/m^2^, respectively, which is similar to that of the asphalt–aggregate interface. Therefore, it can be concluded that the interfacial adhesion strength between asphalt binder and calcium alginate carrier can satisfy the requirements of the asphalt mixture.

**Figure 4:**
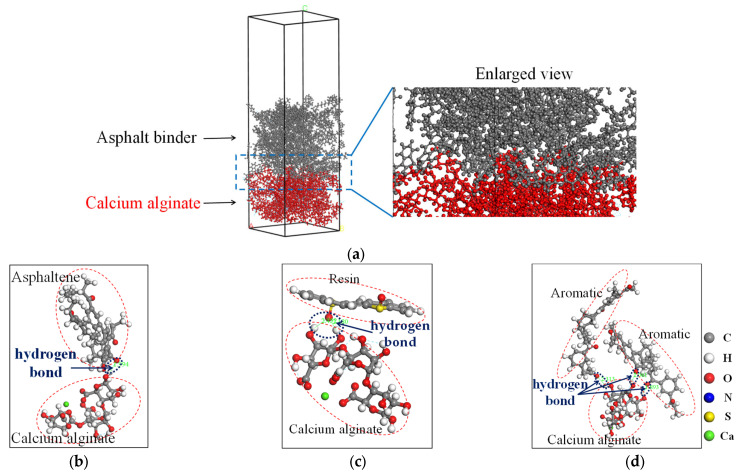
Illustration of the hydrogen bonds between calcium alginate and asphalt binder: (**a**) The interface region; (**b**) Calcium alginate and asphaltene; (**c**) Calcium alginate and resin; (**d**) Calcium alginate and aromatic.

The thermodynamic equilibrium configuration of the interfacial adhesion model of calcium alginate and asphalt binder is shown in Figure 4. The numerous microvoids and folds on the surface of calcium alginate allow asphalt binder to enter its interior through diffusion and permeation, forming good mechanical meshing effects on the interface area and contributing a lot to the improvement of interfacial adhesion strength.

On the other hand, the hydrogen atoms on the hydroxyl group of calcium alginate molecules and the oxygen atoms of asphalt molecules form hydrogen-bond cross-linking in the interface area, enhancing the interaction effect between asphalt binder and calcium alginate. In the aged asphalt binder, the amount of oxygen atoms are increased due to oxidative reactions, and the hydrogen bonds are increased accordingly. In view of this, it is considered that the interfacial adhesion strength can be enhanced by the oxidative aging of asphalt binder. Nevertheless, it was found that if the aging degree increases continuously, some unexpected phenomenon, such as hardening, might occur and adversely affect the interfacial adhesion strength [23]. Therefore, only a certain degree of aging is favored with respect to the interfacial adhesion. The interfacial adhesion work of TiO_2_ with asphalt binder before and after aging is −205.82 mJ/m^2^ and −210.87 mJ/m^2^, respectively, both higher than that of calcium alginate with asphalt binder. It can be concluded that the addition of TiO_2_ can improve the interfacial adhesion strength between calcium alginate carrier and asphalt binder. In addition, the aging of asphalt binder has little effect on its interfacial adhesion work with TiO_2_.

## 3. Establishment of Molecular Models

### 3.1. Molecular Model of Asphalt Binder

**Figure 5:**
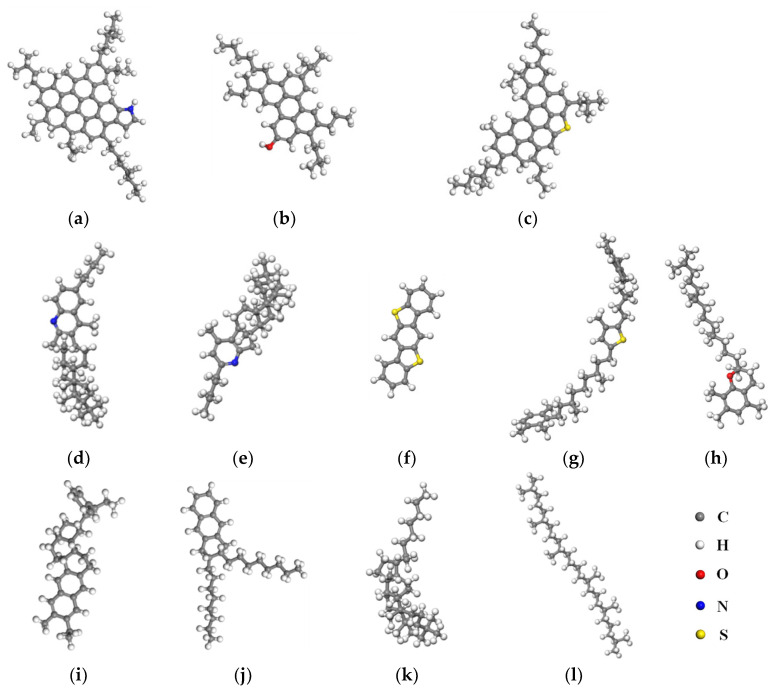
Representative molecular structures of the four components of asphalt binder: (**a**–**c**) Asphaltene; (**d**–**h**) Resin; (**i**,**j**) Aromatic; (**k**,**l**) Saturate.

As an organic mixture, the chemical composition of asphalt binder is very complex, making it very difficult to establish its precise molecular structure [24,25]. Up to now, there are two means to establish the molecular model of asphalt binder, namely the average molecular model and the multi-component molecular model [26,27,28,29]. Research results demonstrate that reliable simulation results can be obtained by using the established molecular models with respect to issues about asphalt modification, regeneration, and interfacial adhesion [30,31,32]. It is proposed that the multi-component molecular model better displays the diversity of the chemical components of asphalt binder and the interaction among the chemical components [33]. Therefore, the four-component molecular model proposed by Li and Greenfield was adopted in this paper [28]. It contains four chemical components: asphaltene, resin, aromatic and saturate, each of which is composed of several representative molecules. As shown in Figure 5, there are three molecules for asphaltene (Figure 5a–c), five molecules for resin (Figure 5d–h), two molecules for the aromatic component, and two molecules for the saturated component.

**Figure 6:**
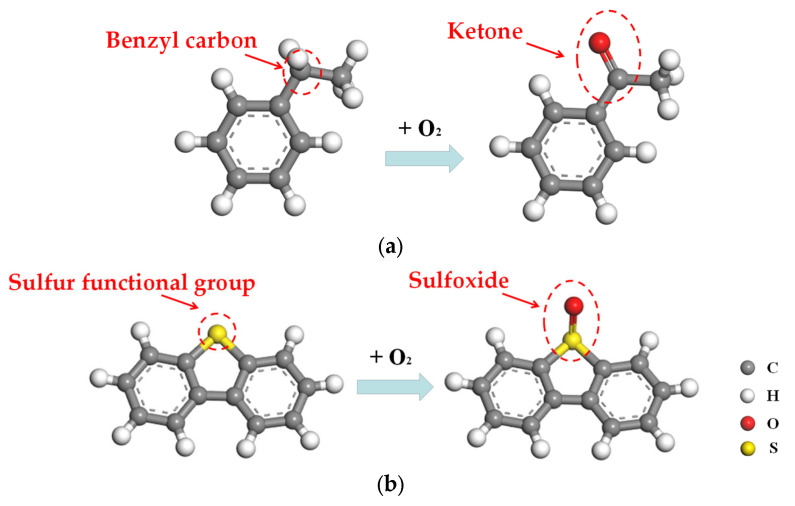
Oxidative aging process: (**a**) Formation of ketone; (**b**) Formation of sulfoxide.

Considering the significant effect of aging on behavior of asphalt binder [34], it is necessary to establish the molecular model of aged asphalt binder. In this paper, the oxidative aging process was simulated by introducing oxygen atoms to the readily oxidizing sites of asphalt molecules. The benzyl carbon and sulfur functional groups are susceptible to oxidation, whose oxidation products are ketone and sulfoxide, respectively [35], as shown in Figure 6.

**Figure 7:**
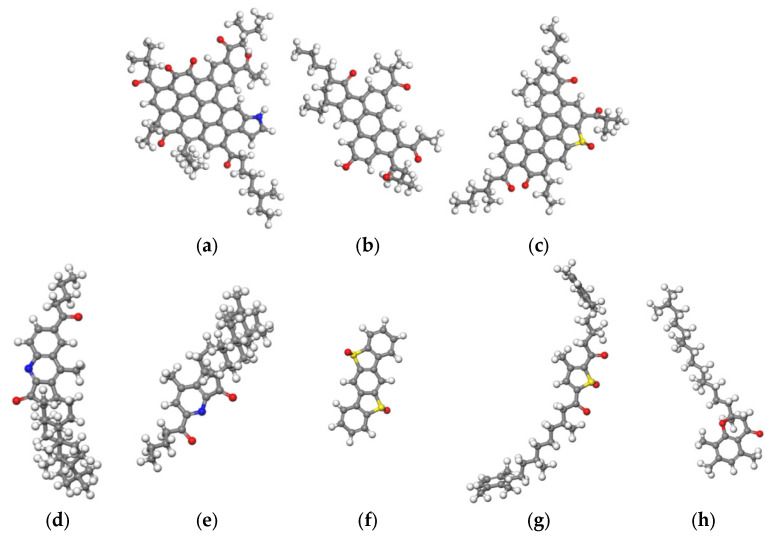

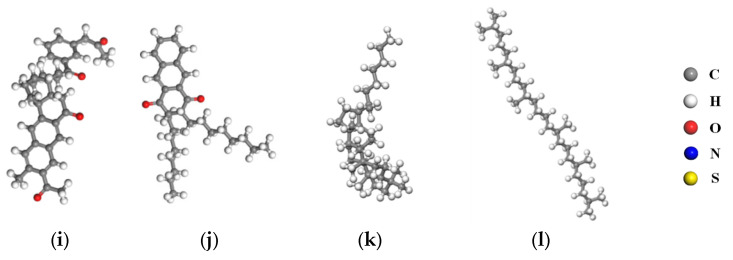
Representative molecular structures of the four components of aged asphalt binder: (**a**–**c**) Aged asphaltene; (**d**–**h**) Aged resin; (**i**,**j**) Aged aromatic; (**k**,**l**) Aged saturate.

After the oxidative reaction of asphalt molecules with oxygen in Figure 6, the representative molecular structures of the four components of aged asphalt binder were obtained as shown in Figure 7. Since saturates are non-polar and do not have readily oxidizing functional groups, the molecular structures of saturates remain unchanged before and after the oxidative aging process [36,37].

**Figure 8:**
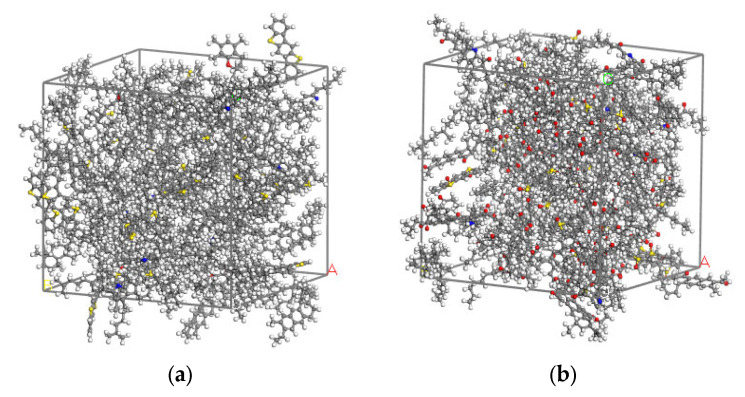
Molecular models of asphalt binder: (**a**) Pre-aging; (**b**) Post-aging.

Details of representative molecules in the pre- and post-aging asphalt binders are listed in Table 4. The representative molecular structures were input into the simulation software MS 7.0, and then put into the amorphous cell according to the details in Table 4. The molecular model of asphalt binder with the lowest energy state was obtained after 5000 steps of geometry optimization in the Forcite module. Then, the dynamics simulation of 100 picosecond (ps) under the canonical (NVT) ensemble and another 100 ps under the isothermal-isobaric (NPT) ensemble was carried out, making the molecular model similar to the real state of asphalt binder under normal temperature and pressure. The snapshot of the final stable molecular model is shown in Figure 8.

### 3.2. Molecular Model of Calcium Alginate Carrier

**Figure 9:**
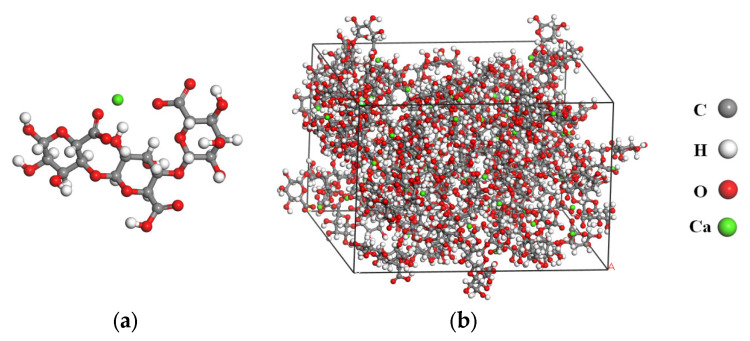
Molecular model of calcium alginate: (**a**) Molecular conformation; (**b**) Molecular model.

According to the authors’ previous research [7], the main component of calcium alginate carrier is calcium alginate and a certain amount of titanium dioxide. The chemical formula of calcium alginate is C_18_H_24_CaO_19_, and its molecular conformation is illustrated in Figure 9a. The amorphous cell model of calcium alginate was established in MS 7.0 software, and structural optimization was performed on it. Then, the dynamics simulation was conducted for 100 ps under NVT Ensemble and another 100 ps under NPT Ensemble, respectively. Finally, the molecular model of calcium alginate with stable conformation was obtained as shown in Figure 9b.

**Figure 10:**
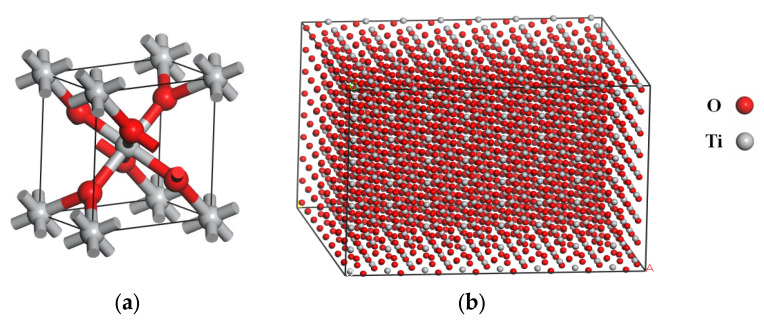
Molecular model of TiO_2_: (**a**) Molecular structure; (**b**) Molecular model.

The molecular model of TiO_2_ is shown in Figure 10a. It was cut along the crystal face (0,0,1), and the thickness was set as 29.6 Å. Then, the configuration was optimized, and the supercell was constructed to enlarge the interface area. Next, it was converted into a 3D model with periodic boundary conditions by adding a vacuum layer with thickness of 0 Å on its surface. Finally, the force field was manually assigned to the atoms in the model, and the ionic bonds were deleted. The established molecular model of TiO_2_ is shown in Figure 10b.

### 3.3. Molecular Model of Interfacial Adhesion Model

**Figure 11:**
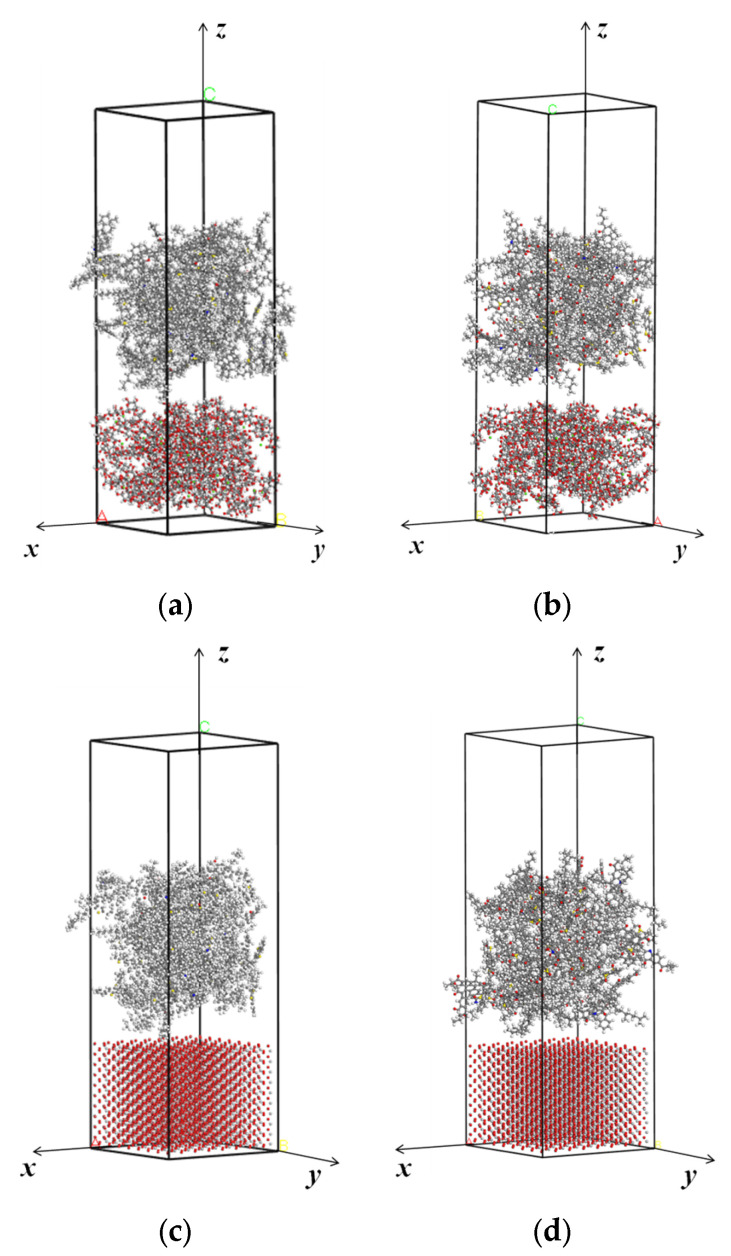
The initial configurations of the interfacial adhesion models: (**a**) Calcium alginate and unaged asphalt binder; (**b**) Calcium alginate and aged asphalt binder; (**c**) TiO_2_ and unaged asphalt binder; (**d**) TiO_2_ and aged asphalt binder.

In order to investigate the interfacial adhesion property between capsule shell and asphalt binder, the interfacial adhesion model was established by superimposing the optimized molecular model of capsule shell and asphalt binder. Shell materials (calcium alginate and titanium dioxide) are solid at ambient temperature and pressure. Their molecules have much higher diffusion ability than the molecules of asphalt binder. Hence, the atoms in the molecular models of shell materials were fixed to increase the computational efficiency. The established interfacial adhesion models are illustrated in Figure 11a–d. The details about the interfacial adhesion models are shown in Table 5.

## 4. Molecular Dynamics Simulation of Interfacial Adhesion Behavior

### 4.1. Simulation Details

**Figure 12:**
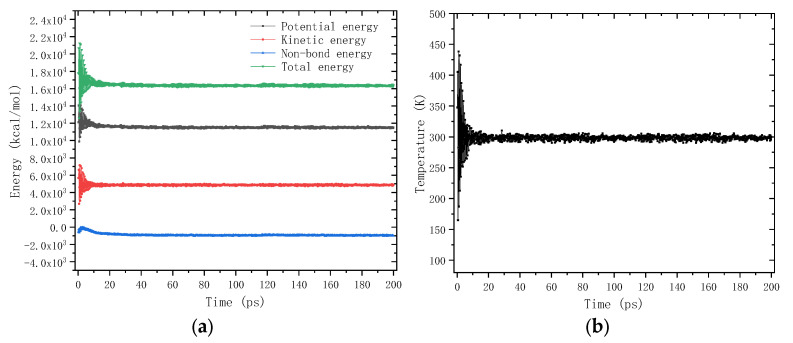
The fluctuation of energy and temperature during the dynamics simulation process: (**a**) Energy; (**b**) Temperature.

The MD simulation was conducted on the interfacial adhesion models through the 5000-step geometry optimization and the 200 ps dynamics simulation under the NVT ensemble. The time step was 1 femtosecond (fs), and the simulation temperature was set as 298.15 K. The energy and temperature of the interfacial adhesion model varied with the simulation time. Typically, it is considered that the thermodynamic equilibrium state is achieved when the percent amplitude of fluctuation is less than 10% [38]. Taking the interfacial adhesion model of titanium dioxide and asphalt binder as an example, as shown in Figure 12, the energy and temperature reached the setting value at 30 ps, and then fluctuated slightly near the setting value. The percent amplitude of fluctuation of energy was 5.16%, and that of temperature was only 1.03%. Therefore, the 200 ps simulation time was enough to allow the system to reach a thermodynamic equilibrium state.

### 4.2. Thermodynamic Equilibrium State

**Figure 13:**
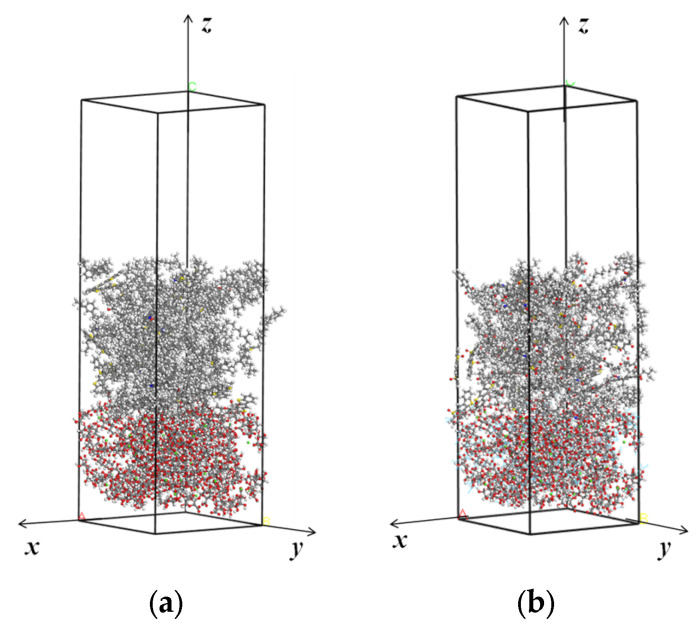

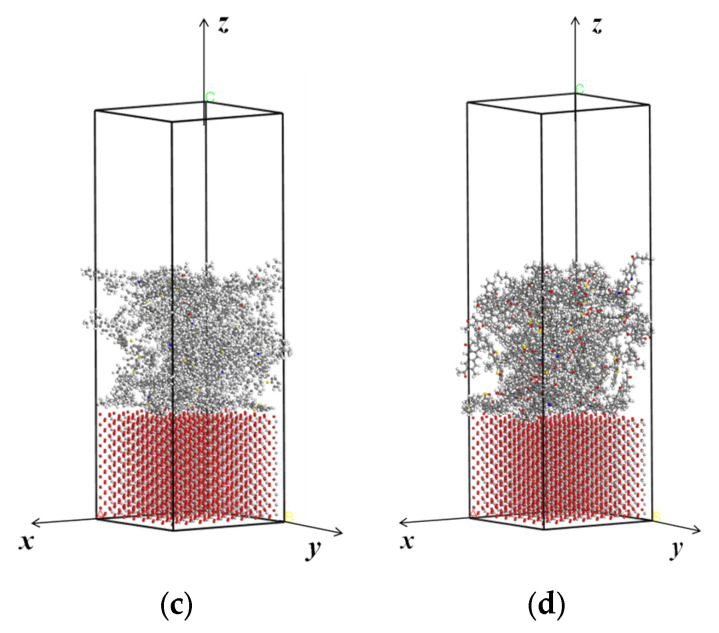
The thermodynamic equilibrium state of the interfacial adhesion models: (**a**) Calcium alginate and unaged asphalt binder; (**b**) Calcium alginate and aged asphalt binder; (**c**) TiO_2_ and unaged asphalt binder; (**d**) TiO_2_ and aged asphalt binder.

Figure 13 shows the thermodynamic equilibrium state of the interfacial adhesion models. It can be seen that after 200 ps dynamics simulation, asphalt binder moved towards the shell materials, ultimately adsorbing on the surface of shell material. It demonstrates that asphalt molecules and calcium alginate carrier are attracted to each other.

## 5. Conclusions

In this study, the interfacial adhesion property between calcium alginate carrier and asphalt binder was investigated by constructing the molecular model of interfacial adhesion and conducting MD simulations. The data during the simulation process was extracted and analyzed to characterize and evaluate the interfacial adhesion property. The main conclusions are as follows:

(1) Asphalt binder moved towards the calcium alginate carrier during the simulation period and finally adsorbed on its surface, indicating that they were compatible with each other.
(2) Asphalt binder wetted the surface of the calcium alginate carrier, and permeated into the porous structure of calcium alginate, forming a good interface interaction effect. Two indexes, namely permeation depth and permeation degree, were proposed to quantitatively characterize the permeation behavior of asphalt binder into calcium alginate carrier.
(3) It was very hard for asphalt molecules to infiltrate into the interior of TiO_2_, and thus asphalt molecules could only accumulate and spread on the surface of TiO_2_.
(4) The interfacial adhesion strength between asphalt binder and calcium alginate carrier can satisfy the requirements of the asphalt mixture. In addition, the formation of interfacial adhesion strength between asphalt binder and calcium alginate carrier mainly depended on the Van der Waals interactions, followed by electrostatic interactions.
(5) The interfacial adhesion property could be further improved by a certain degree of asphalt aging and the addition of titanium dioxide in the calcium alginate carrier.

In conclusion, calcium alginate is a suitable material for serving as the carrier of asphalt rejuvenator in terms of its excellent interfacial adhesion property with asphalt binder. In addition, TiO_2_ is recommended to be added in calcium alginate carrier to further improve its compactness and its adhesion strength with asphalt binder. In the following research, different aging levels of asphalt binder will be considered, and the actual working process of the calcium alginate capsule will be simulated and observed to investigate its working mechanism.

## Figures and Tables

**Table 1:** Calculation results of spreading coefficient.

Shell Material	Asphalt Binder	*W_adhesion_* (mJ/m^2^)	*W_cohesive_* (mJ/m^2^)	*S* (mJ/m^2^)
Calcium alginate	Unaged	−114.18	−80.26	42.19
Titanium dioxide	Unaged	−205.82	−80.26	125.56
Calcium alginate	Aged	−186.37	−83.41	102.96
Titanium dioxide	Aged	−210.87	−83.41	127.46

**Table 2:** Permeation depth and permeation degree of the four components of asphalt binder.

Interface Type	Permeation Depth (Å)				Permeation Degree (Å)			
	Asp ^1^	Res ^2^	Aro ^3^	Sat ^4^	Asp	Res	Aro	Sat
Calcium alginate–unaged asphalt binder	4.9	8.3	7.8	10.4	5.5	10.4	7.9	31.6
Titanium dioxide–unaged asphalt binder	0	0	0	0	0	0	0	0
Calcium alginate–aged asphalt binder	6.6	11.8	12.8	11.8	13.7	14.5	18.0	33.9
Titanium dioxide–aged asphalt binder	0	0	0	0	0	0	0	0

^1^ Asp refers to Asphaltene; ^2^ Res refers to Resin; ^3^ Aro refers to Aromatic; ^4^ Sat refers to Saturate.

**Table 3:** Calculation results of the interfacial adhesion energy/work.

Interface Type	∆*E_valence_* ^1^ (kcal/mol)	∆*E_vdW_* ^2^ (kcal/mol)	∆*E_elec_* ^3^ (kcal/mol)	∆*E_adhesion_* (kcal/mol)	*W_adhesion_* (mJ/m^2^)
Calcium alginate–unaged asphalt binder	0	−214.83	−21.56	−236.39	−114.18
Titanium dioxide–unaged asphalt binder	0	−398.26	−27.85	−426.11	−205.82
Calcium alginate–aged asphalt binder	0	−204.79	−181.50	−386.29	−186.37
Titanium dioxide–aged asphalt binder	0	−405.05	−32.01	−437.06	−210.87

^1^ ∆*E_valence_* represents the contribution of bonding interaction energy to ∆*E_adhesion_*; ^2^ ∆*E_vdW_* represents the contribution of van der Waals interaction energy to ∆*E_adhesion_*; ^3^ ∆*E_elec_* represents the contribution of electrostatic interaction energy to ∆*E_adhesion_*.

**Table 4:** Details of the representative molecules of pre- and post-aging asphalt binders.

Chemical Component	Code	Number	Pre-Aging		Post-Aging	
			Chemical Formula	Molecular Weight (g/mol)	Chemical Formula	Molecular Weight (g/mol)
**Asphaltene**	a	2	C_66_H_81_N	888.4	C_66_H_67_NO_7_	986.3
	b	3	C_42_H_54_O	574.9	C_42_H_46_O_5_	630.8
	c	3	C_51_H_62_S	707.1	C_51_H_54_O_5_S	779.0
Resin	d	4	C_40_H_59_N	553.9	C_40_H_55_NO_2_	581.9
	e	4	C_36_H_57_N	503.9	C_36_H_53_NO_2_	531.8
	f	15	C_18_H_10_S_2_	290.4	C_18_H_10_O_2_S_2_	322.4
	g	4	C_40_H_60_S	573.0	C_40_H_56_O_3_S	616.9
	h	5	C_29_H_50_O	414.7	C_29_H_48_O_2_	428.7
Aromatic	i	11	C_35_H_44_	464.7	C_35_H_36_O_4_	520.7
	j	13	C_30_H_46_	406.7	C_30_H_42_O_2_	434.7
Saturate	k	4	C_35_H_62_	482.9	C_35_H_62_	482.9
	l	4	C_30_H_62_	422.8	C_30_H_62_	422.8

**Table 5:** Details about the interfacial adhesion models.

Interface Type	Size (Å^3^)	N_asphalt_^1^	N_shell_^2^	N_sum_^3^
Calcium alginate-unaged asphalt binder	37.93 × 37.93 × 122.29	5572	3720	9292
Calcium alginate-aged asphalt binder	37.95 × 37.95 × 124.29	5472	3720	9192
Titanium dioxide-unaged asphalt binder	37.93 × 37.93 × 115.69	5572	3840	9412
Titanium dioxide-aged asphalt binder	37.95 × 37.95 × 115.96	5472	3840	9312

^1^ N_asphalt_ represents the number of atoms contained in asphalt binder; ^2^ N_shell_ represents the number of atoms contained in shell material; ^3^ N_sum_ represents the total number of atoms contained in the interfacial adhesion model.

## Data Availability

The research data is available by contacting the corresponding author.
